# The Effects of a High Carbohydrate Diet Combined with High Molecular Weight Carbohydrate Supplementation on Anaerobic Performance and Oxidative Stress in Elite Swimmers

**DOI:** 10.3390/jcm14113846

**Published:** 2025-05-30

**Authors:** Mateusz Gawełczyk, Sławomir Jagsz, Adam Zając, Józef Langfort

**Affiliations:** Institute of Sport Sciences, The Jerzy Kukuczka Academy of Physical Education in Katowice, 40-065 Katowice, Poland; m.gawelczyk@awf.katowice.pl (M.G.); s.jagsz@awf.katowice.pl (S.J.); j.langfort@awf.katowice.pl (J.L.)

**Keywords:** Wingate test, swimming performance, oxidative stress, elite swimmers, glycogen, sports nutrition

## Abstract

**Background/Objectives**:Training periodization is fundamental to optimizing athletic performance, with carbohydrate metabolism playing a critical role in supporting high-intensity efforts by facilitating muscle glycogen resynthesis. Recent studies suggest that high carbohydrate diets and high molecular weight carbohydrate (HMWC) supplementation can improve both endurance and anaerobic performance, while potentially influencing oxidative stress. This study investigates the effects of a high carbohydrate diet combined with HMWC supplementation on anaerobic performance and oxidative stress markers in elite swimmers. **Methods**: Eight national-level swimmers (tier 3–4) completed a three-day training microcycle with dietary interventions. Anaerobic capacity was assessed using Wingate tests for upper and lower limbs, while swimming performance was evaluated through an 8 × 100 m exercise protocol. The study was conducted using a one group quasi-experimental design with a pre-test/post-test structure, with participants acting as their own controls. Baseline measurements were taken prior to the intervention, followed by the administration of the high carbohydrate diet and HMWC supplementation. Post-intervention assessments were performed using the same test protocols to assess changes in performance and oxidative stress markers (such as GSH, CK, MDA, FRAP), which were determined by ELISA. The samples were stored at −80 °C until the evaluations. STATISTICA 5.0 (StatSoft, Inc., 1995) was used for statistical analysis of the obtained results. **Results**: The obtained results demonstrated significant improvements in peak power output for the lower limbs following supplementation (*p* < 0.001) and a reduced time to peak power for the upper limbs (*p* < 0.001). Additionally, velocity during the final swimming segments increased significantly following the intervention (*p* < 0.001). However, no notable changes were observed in antioxidant enzyme activity (SOD, CAT, GPx, GR) or low molecular weight antioxidants, suggesting a potential ceiling effect in redox adaptations. Lipid peroxidation, measured by MDA levels, increased post-supplementation (*p* < 0.05), indicating oxidative stress associated with high-intensity training and supplementation. **Conclusions**: The findings underscore the efficacy of combined dietary strategies with HMWC in enhancing anaerobic performance in swimming, while highlighting the necessity for further exploration of oxidative stress dynamics.

## 1. Introduction

Training periodization is a systematic approach to training that divides an athlete’s training schedule into specific blocks, each with distinct goals and purposes [1]. Of the two available sources of glucose during exercise, i.e., glucose derived from blood and intramuscular muscle glycogen breakdown, the later plays a pivotal role in ATP resynthesis at higher exercise intensities [2,3]. The main objective of periodization is to progressively improve an athlete’s performance while minimizing the risk of injury as well as overreaching and overtraining. Coaches and competitive athletes use various periodization strategies to optimize the effects of training in order to reach peak performance for a particular season, such as the national championships, European Championships, World Championships, or the Olympic Games [4,5,6]. Recently, some research has focused on environmental factors which implemented into a mesocycle could bring additional benefits to the traditional training procedures and thus further improve exercise capacity and performance. Such an approach has been recognized with a high carbohydrate diet or carbohydrate supplementation that improves muscle glycogen resynthesis after exercise [7,8], allowing improved work performance at high intensity [9,10,11].

The most recent studies of dietary carbohydrate intake and exercise capacity clearly indicate that availability and utilization of this fuel can profoundly improve the performance of both endurance exercise and endurance training [12,13]. The relative contribution of carbohydrate metabolism is primarily determined by the intensity and duration of exercise. In recent years, some studies have documented that carbohydrates are also the primary fuel for anaerobic metabolism during high intensity exercise [14]. Importantly, the anaerobic metabolic system can influence swimmers’ performance over short distances, e.g., 50 and 100 m [15,16]. However, a recent study showed that neither a high carbohydrate, low fat diet nor a low carbohydrate, high fat diet affected exercise performance up to 70% VO_2_max [17]. However, among scientists, coaches, and athletes there are still numerous controversies regarding carbohydrate intake before, during, and after exercise.

Currently, the most used training approach of competitive swimmers includes the “strength and conditioning training”, which is based on dry-land and under-water exercises [18,19]. Energy production during the high intensity exercises is derived from the degradation of intra-muscular phosphocreatine and glycogen stores (anaerobic metabolism) [14]. Prolonged periods of anaerobic efforts drain muscle glycogen stores, leading to a decrease in power output and a reduction in general work rate during training and competition that can also lead to fatigue [20].

Rates of post-exercise glycogen resynthesis differ depending on the type of consumed carbohydrate. Recently, the ingestion of high molecular weight carbohydrate (HMWC) was found to provide superior benefits in relation to low molecular weight carbohydrate (LMWC) on performance. Indeed, Piehl Aulin et al. [21] reported a markedly higher rate of muscle glycogen synthesis after ingesting drinks containing a polyglucoside as compared to drinks containing monomers and oligomers. In other studies, it has been proven that an intake of HMWC solution as compared to the same volume of isoenergetic LMWC solution caused a 167% increase in muscle glycogen synthesis after the end of physical exercise, which caused muscle glycogen depletion [21,22].

During high contractile activity, skeletal muscle overproduces reactive oxygen species (ROS) compared to levels found at rest [23,24]. Studies on rats showed that production of reactive oxygen species could be related to diminished glycogen content in both anaerobic and aerobic muscle fibers [25]. This means that in conditions of reduced glycogen levels, the cytosolic pathway is the primary source of free radical production. In line with this postulate, there are studies which indicate that the lack of glycogen phosphatase in patients with McArdle disease leads to the activation of the cytosolic pathway of free radical production catalyzed by xanthine oxidase [26]. A similar effect has been observed in patients with Pompe disease, in which the deficiency of another enzyme involved in glycogen hydrolysis, alpha-glucosidase, also causes a strong induction of oxidative stress [27]. As a protective mechanism, however, both physical training and exercise induces antioxidant production, which have been related to a reduction in free radical concentration. These systems include various antioxidant enzymes, e.g., glutathione peroxidase (GPx), glutathione reductase (GR), superoxide dismutase (SOD) and catalase (CAT), and low molecular weight antioxidants, e.g., tocopherols and reduced glutathione (GSH) that act mostly in lipophilic and hydrophilic milieus, respectively [28,29].

The antioxidant response to oxidative stress resulting from regular physical activity in professional athletes has been widely studied over the past few years. However, most studies have focused on this issue in endurance sport disciplines [29]. There is a gap in the research undertaken in relation to anaerobic exercise. Data on the effects of anaerobic exercise combined with supplementation of HMWC solution on antioxidant stress in swimmers are scarce.

In modern competitive sports, one of the most difficult challenges for coaching teams is maintaining an athlete’s form between key competitions. Depending on the sport discipline, the athlete can have one major competition during the season, such as the Olympic Games, or he competes in a series of competitions during which he must maintain high or peak performance. Such is the case in team sport games, where athletes at the highest sports level must maintain a high level of performance throughout the entire season and perhaps peak for the playoffs or the finals. In most individual sport disciplines at the elite level, athletes need to reach a high level of performance to qualify for the major competitions and then during the main competitions such as the European, World Championships, or the Olympic Games, when they must peak at the time of the finals in a particular event. In the period between important competitions, to maintain the athlete’s current form, sustaining mesocycles are used within the frame of training periodization.

The present study investigated the effects of a combined high carbohydrate diet and HMWC supplementation on anaerobic performance and selected indices of pro-oxidant/antioxidant balance. We hypothesize that a high carbohydrate diet and HMWC supplementation will reduce oxidative stress. In addition, we hypothesize that a high carbohydrate diet and HMWC supplementation will reduce lipid peroxidation, which is a marker of oxidative stress.

## 2. Materials and Methods

### 2.1. Participants

Eight national-level (tier 3–4) healthy male swimmers participated in the study. The age of the study participants equaled 20 ± 0.5 years, body height of 1.87 ± 0.51 m, and a body mass of 77.0 ± 10.6 kg (mean ± SD). The inclusion criteria were (a) minimum 8 years of experience in competitive sports training, with at least 8 training sessions per week; (b) age above 18 years old; and (c) no use of dietary supplements in the 10 weeks prior to the experiment. The examined group consisted of four athletes specializing in freestyle, two in butterfly, and one in backstroke and breaststroke. The study was conducted according to the Declaration of Helsinki with the research procedure approved by of the University Bioethics Committee at the Jerzy Kukuczka Academy of Physical Education in Katowice (5/2005). Written consent was obtained from all participants.

### 2.2. Experimental Design

**Figure 1:**
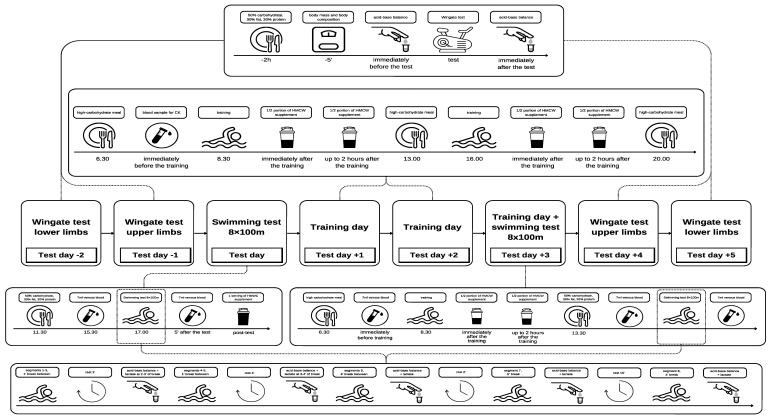
Experimental design.

The study was conducted using a one-group quasi-experimental design with a pre-test/post-test structure, with participants acting as their own controls. Baseline measurements were taken prior to the intervention, followed by the administration of the high carbohydrate diet and HMWC supplementation. Participants carried out 6 separate physical fitness tests: 4 Wingate tests (2 for upper and 2 for lower limbs) and 2 swimming tests (Figure 1). The study was divided into three phases—pre-test (before supplementation), training days with the prescribed diet and supplementation, and post-test (following the intervention). The first two Wingate tests (phase 1) for the upper and lower limbs were conducted three and two days prior to the three-day microcycle (phase 2). On the day prior to the start of the microcycle, the athletes performed an 8 × 100 m swim test [30], conducted in a 25 m pool. After the three-day microcycle (phase 2), the swimmers repeated the procedure used in a reverse order (phase 3).

On the experimental day, the swimmers arrived at the laboratory at 8:30 a.m. to perform two sets assessing anaerobic capacity (Wingate test) on separate days for the upper and lower limbs, respectively, 3 days and 2 days prior to the microcycle, and 2 and 3 days after its completion. Before starting the anaerobic capacity tests, height and body mass measurements were taken for all participants. Before and after cessation of each Wingate test, capillary blood lactate concentration and acid–base balance variables were assessed.

### 2.3. Experimental Protocol

#### 2.3.1. Wingate Test

Prior to the 30 s Wingate test (Excalibure Sport ergometer, Lode, Groningen, The Netherlands) for the upper (4% B.W.) and the lower (7.5% B.W.) limbs, the subjects performed a 3 min warm-up by working separately the lower or upper limbs on the cycloergometer (50 W) followed by 6 min of passive rest. Before the first Wingate test, each participant adjusted the position of the seat according to their anthropometric features. This position was replicated for the second Wingate test. Participants were motivated verbally by the examiner during each Wingate test.

During the Wingate test the following variables were recorded: relative (PPrel) and absolute maximum power (PP), mean power (MP), minimum power (MinP), total work done (TW), time to peak power (TPP), and fatigue slope (FS). To determine the efficiency of glycolysis, blood lactate (LA) concentrations and blood acid–base balance indices were evaluated.

#### 2.3.2. Swimming Test

The swimming test was conducted at approximately 5:00 p.m., which is the customary time for swimming competition finals. Prior to the commencement of the test, the athletes engaged in a standardized in water warm-up routine, which was followed by the 8 × 100 m step test in a 25 m pool, in accordance with the protocol proposed by Maglischo [30]. The test consists of a series of 100 m progressive swims with controlled rest intervals and evaluation of post-exercise blood lactate concentration. The protocol consists of the following series:

3 × 100 m at 75% max (1 min, then 3 min rest interval; blood sampling at 2–3 min);
2 × 100 m at 85% max (1 min and 4 min rest interval; blood sampling at 3–4 min);
1 × 100 m at 90% max (6 min rest interval; blood sampling at 4–5 min post-exercise);
1 × 100 m at 95% max (20 min rest interval; blood sampling at 5–6 min post-exercise);
1 × 100 m at 100% max (blood samples drawn at 4 min post-exercise).

During the swimming test, 50 µ of capillary blood samples from the fingertip were collected to determine acid–base balance variables and LA concentration during the designated rest period following the completion of each segment of the 8 × 100 m swimming trial.

### 2.4. Nutrition and Supplementation

Subjects were instructed to abstain from alcohol and caffeine for a minimum of 72 h prior to each trial, with a minimum interval of 72 h between trials. Each time it was checked using a checklist. The pre- and post-exercise trials were conducted at the same time of day to maintain similar experimental conditions. A standardized breakfast, comprising 50% carbohydrates, 30% fats and 20% proteins (approximately 4.5 kcal/kg body weight), was provided for consumption two hours prior to the Wingate test. A standardized meal, consisting of 50% carbohydrates, 30% fats, and 20% proteins (approximately 6.5 kcal/kg body weight), was also provided for participants to consume three and a half hours prior to attending the swimming test trial. Between the tests, the athletes took part in a controlled training process.

Immediately after the first swimming test, participants were provided with a high molecular weight carbohydrate supplement. The supplement comprised the following ingredients: hydrolyzed starch (of high molecular weight), citric acid, sodium carbonate, magnesium salts of citric acid, potassium citrate, calcium salts of citric acid, and a vitamin blend (L-ascorbic acid, nicotinamide, D-calcium pantothenate, thiamine hydrochloride). The supplement also contained vitamin B2 (riboflavin), vitamin B6 (pyridoxine hydrochloride), vitamin B1 (thiamine hydrochloride), vitamin B5 (pantothenic acid), vitamin B7 (biotin), vitamin B9 (folic acid), vitamin B12 (cyanocobalamin), flavoring agents, and coloring agents, including beetroot extract or beta-carotene, depending on the flavor. Additionally, the supplement included a sweetening agent, stevia glycosides. Each 37.5 g serving of the supplement provided 33.75 g of HMWC, with an accompanying fat content of less than 0.1 g and no protein. Furthermore, each serving provided 200% of the recommended daily intake of vitamin C and 20% of vitamins B2, B3, B5, B6, B9, B12, and biotin.

The carbohydrate supplement was consumed in quantities adjusted to the athlete’s body weight, with the objective of supplementing their daily intake of carbohydrates from meals. On days one and two of the controlled diet during the three-day microcycle, the meals contained 300 g of carbohydrates, while on day three, this amount was increased to 350 g. On the first two days, HMWC provided about 46% of total carbohydrate (~285 g) and on the last day 39% (~239 g). This was carried out to achieve a level of 8 g/kg of CHO. Protein provided 15% of total energy expenditure. The remaining calories needed to achieve a neutral energy balance of the diet were provided from dietary fat. The recommended intake of carbohydrates per body weight per day, as outlined by Maglischo [30], was adhered to. The athletes lived on an academic campus for the duration of the study, and all meals were provided by the university’s canteen. The liquid supplement was consumed in two portions: half immediately following each training session, and the remaining portion (prepared in bottles) within two hours of the training session. The final dose of the supplement was taken after the morning water training session on the day of the second swimming test. The study participants were asked to record all meals, snacks, and drinks taken during the study.

### 2.5. Biochemical Analysis

Before the first and after the third Wingate test, 50 µL of capillary blood from the fingertip was drawn to determine lactate concentration and acid–base balance (GEM Premier 3000 analyzer, Werfen, Barcelona, Spain). Prior to and following the swimming performance tests, 7 mL of blood was drawn from the participants’ cubital vein for the purpose of determining creatine kinase (CK) activity, uric acid (UA), superoxide dismutase (SOD), catalase (CAT), glutathione peroxidase (GPX), malondialdehyde (MDA), ferric-reducing antioxidant power (FRAP), and isoenzymatic profile of lactate dehydrogenase in fresh plasma samples.

Fresh whole blood samples were immediately assayed for reduced glutathione (GSH) by the colorimetric method using 5,5′-dithiobis-2-nitrobenzoic acid [31]. A portion of heparinized blood was centrifuged at 1000× *g* for 10 min at 4 °C to separate plasma and erythrocytes, which were then washed three times with cold saline (4 °C) and kept frozen at −80 °C (for a period not exceeding one month, without repeated freezing and thawing) until assayed for the activities of erythrocyte antioxidant enzymes, i.e., superoxide dismutase (SOD, EC 1.15.1.1) with the commercial kit RANSOD SD125 (Randox Laboratories Ltd., Crumlin, UK); glutathione peroxidase (GSH-Px, EC 1.11.1.9) with the commercial kit RANSEL RS505 (Randox Laboratories Ltd., Crumlin, UK); catalase (CAT, EC 1.11.1.6) according to the method of Aebi [32]; and glutathione reductase (GR, EC 1.6.4.2) according to Glatzle et al. [33]. The activities of all antioxidant enzymes were measured at 37 °C and expressed per 1 g of hemoglobin as determined by a standard cyanmethemoglobin method using a diagnostic kit No. HG980 (Randox Laboratories Ltd., Crumlin, UK). Fresh plasma samples were assayed for uric acid (UA) concentration using diagnostic kits from Randox Laboratories (UA230). Total plasma antioxidant capacity was assessed by the ferric-reducing ability of plasma (FRAP) assay according to Benzie and Strain [34]. Biochemical analyses were performed in a certified laboratory, which meets the requirements of PN-EN ISO 9001:2015 (certificate nr. PW-19912-18B) [35], according to the instructions provided by the manufacturers of the laboratory tests used in this study.

### 2.6. Statistical Analysis

Data were analyzed using STATISTICA 5.0 (StatSoft, Inc., Tulsa, OK, USA, 1995). A *p*-value of less than 0.05 was used to determine statistical significance. Results are presented as means (x), standard deviations (±SD), and standard errors (±SE). The normality of the distributions of the study variables was tested using the Shapiro–Wilk test. A paired samples t-test was used to examine the mechanical variables of the Wingate test and swimming speed in the swimming test to determine the effect of supplementation conditions on participants’ exercise capacity. The effect size was calculated using Cohen’s d for t-test. The effect size is described as small when *d* = 0.2, *d* = 0.5 for a medium effect, and *d* = 0.8 for a large effect. Multivariate analysis of variance (ANOVA) with a post hoc comparison of means using the Tukey test for samples of equal size was performed to determine whether there were differences in blood markers before and after supplementation. The level of statistical significance was set at *p* ≤ 0.05. The strength of the effect was calculated using the eta-squared measure, which describes the ratio of the variance of the dependent variable explained (in a purely correlational sense) by the independent variable (predictor) at constant values of the other independent variables (predictors), where SSB—intergroup sum of squares and SST—total sum of squares. The strength of the effect is described as small if the eta-squared is <0.059, as medium if it is 0.06–0.137, and as large if it is >0.137.

### 2.7. Limitations

This study has several limitations that must be considered when drawing conclusions and considering the implementation of a nutritional supplementation strategy in other athletes. Firstly, the small sample size due to the specific group of athletes with a very specific problem to solve—national and international level swimmers attempting to maintain peak performance between major competitions. Secondly, the lack of a control or placebo group, as it was not possible to split the athletes, who must follow a very specific training and nutritional periodization program, and whose coaches and medical staff should do everything possible to prepare them for competition. Thirdly, the lack of muscle biopsies made it impossible to determine muscle glycogen stores and their changes following the nutritional supplementation strategy. Fourthly, the short duration limited the study. The nutritional-training intervention was very short due to a specific moment in the season when the athletes had a small gap between major competitions. Furthermore, the aim of the study was to determine if a specific high carbohydrate diet accompanied by high molecular weight carbohydrate supplementation could help athletes maintain peak performance at this very particular time of the competitive season.

## 3. Results

There was a significant increase in both relative and absolute peak power after supplementation for the lower body compared to the pre-supplementation (*p* < 0.001; Table 1). However, no statistically significant difference was observed for the upper body. A notable decrease in the time to peak power was observed following supplementation for the upper body in comparison to the pre-supplementation period (*p* < 0.001; Table 1), whereas no significant difference was noted for the lower body. Following supplementation, a significant increase in the rate of fatigue was observed for the lower body in comparison to the pre-supplementation period (*p* < 0.001; Table 1). However, no significant difference was evident for the upper body. No differences were identified in total work performed for both the upper and lower body, irrespective of supplementation, as well as for work performed to peak power, following peak power and for mean power between the pre- and post-supplementation periods for both the upper and lower body.

A significant increase in LA concentration was observed following the Wingate test, both for the upper and lower limbs, in comparison to the baseline conditions (*p* < 0.05; Table 2). No significant differences were observed in LA concentration between conditions for either the upper or lower body, irrespective of supplementation. A significant reduction in pH and SB was observed following the Wingate test for both the upper and lower body, in comparison to the baseline conditions before and after the supplementation period (*p* < 0.05; Table 2). Following the dietary intervention, there was a significant reduction in SB at rest prior to the Wingate test for both the upper and lower limbs (*p* < 0.05; Table 2). However, after the Wingate test, no significant difference was observed. No significant differences were noted after supplementation in pH for both the resting and post-Wingate test conditions for both the upper and lower limbs.

Following the supplementation period, a notable enhancement in velocity was evident for the last two swims (7 and 8) out of 8 segments, which comprised the swimming test (*p* < 0.001; Table 3). Nevertheless, the discrepancy in mean velocity remained consistent irrespective of the conditions.

Following the swimming test, there was a significant increase in glucose and LA concentration in comparison to baseline condition for both tests (*p* < 0.05; Table 4). No significant differences were observed in glucose and LA concentration for either the resting or post-exercise test periods, regardless of supplementation status. A significant increase in UA was observed following the test period prior to supplementation (T1) in comparison to baseline conditions (*p* < 0.05; Table 4). Nevertheless, no statistically significant difference was observed between T1 and T2. Following the supplementation period, the level of UA after the test was significantly lower in comparison to the pre-supplementation period (*p* < 0.05, effect size = 0.1; Table 4). Following the swimming test, a significant decrease in pH, SB, and BE was observed in comparison to baseline conditions for both tests (*p* < 0.05; Table 4). No significant differences were observed in pH, SB, and BE for either the rest or after the swimming test condition, irrespective of supplementation. No notable changes were observed in the levels of any of the enzymatic antioxidants (GPx, SOD, GR, CAT), reduced glutathione, and lactate dehydrogenase following the test, in comparison to the baseline condition. This was evident both in the pre-supplementation test and the post-supplementation test. A statistically significant reduction in FRAP was observed following the test in the post-supplementation condition in comparison to the pre-supplementation condition (*p* < 0.05, effect size = −0.13; Table 4). However, no significant differences were observed in baseline conditions. A significant increase in MDA was noted before (*p* < 0.05, effect size = 0.12; Table 4) and after (*p* < 0.05, effect size = 0.06; Table 4) the test on post- compared to pre-supplementation conditions. Following the swimming test, there was a significant increase in creatine kinase (CK) activity in comparison to the baseline conditions for both tests (*p* < 0.05; Table 4). Furthermore, a significant increase in CK activity in T2 was observed in comparison to T1 (*p* < 0.05, effect size = 0.14; Table 4).

## 4. Discussion

Carbohydrate oxidation constitutes an important source of energy production for working muscles. Utilization of this substrate increases progressively from low intensity aerobic exercise of about 15% VO_2_max of total energy production to around 100% at intensities close to VO_2_max [36]. Of the two available sources of carbohydrate during exercise, i.e., glucose derived from blood and intramuscular muscle glycogen breakdown, the later plays a pivotal role in ATP resynthesis at higher exercise intensities [37]. Our sustaining mesocycle was a part of block periodization with a specific focus on development of strength and speed. This type of periodization allows for more intense training units followed by short periods of rest and recovery [38]. Our training intervention consisted of in-water resistance exercises, swim training with paddles, tethered swimming, and dry-land resistance exercises, which all are known to improve swimming performance [19,39].

Under these conditions, anaerobic energy delivery for working muscles is crucial for avoiding overtraining and especially for reaching peak performance [40]. Thereby, athletes implement various high carbohydrate diet regimens into mesocycles to increase pre-exercise glycogen levels before the competition or/and for better body recovery after training. Recently, evidence has emerged indicating that the consumption of HMWC supplements promote a more effective way of reaching elevated muscle glycogen levels over pre-exercise values as compared to low weight carbohydrate intake. Benefits of ingestion of HMWC for enhanced exercise capacity have been documented in different types of exercise and in different sport disciplines [41,42,43]. In our study we supplemented swimmers who had just finished competing in the Polish National Championships and were preparing for the European Championships.

The Wingate test is a classic laboratory tool, most often used to assess anaerobic performance and to determine peak and mean power variables. In our study, participants performed both upper and lower limb Wingate tests. It is generally accepted that in swimming, both the upper and lower limbs contribute significantly to sports performance, with the input of the upper limbs being dominant and amounting to 85% of the swimming thrust [44]. The key finding of our study is that HMWC supplementation combined with a high carbohydrate diet has a positive effect on lower limb exercise performance during the 30 s Wingate test. To the best of our knowledge, this is the only study to examine the impact of HMWC supplementation on anaerobic performance evaluated by the Wingate test alone. Previous research has shown positive effects on anaerobic performance during subsequent repeated maximal explosive resistance exercise [42]. Grijota et al. [45] demonstrated no effect of supplementation with 30 g of cyclodextrin on Wingate test performance. Nevertheless, it is important to acknowledge that the experiment was conducted following a series of other studies and a low dosage of the supplement was administered. This may have had an effect on the obtained results [45].

Several studies have shown a strong correlation between Wingate test results and sprint swimming performance. Mean and peak power output from the Wingate test is significantly related to swimming speed in short-distance events such as the 50 m freestyle [46]. This relationship is evident in both upper- and lower-body anaerobic power assessments, indicating that the Wingate test can effectively predict performance in sprint swimming events [47,48,49,50,51]. The findings of this study demonstrate that the participants achieved higher peak power performance during the lower limb Wingate test, both in relative and absolute values. However, the upper limbs showed a shorter time to peak power. It is well documented that the lower limbs play a critical role in starts and turns [52,53,54,55,56], whereas the muscles of the upper limbs contribute to a greater extent than the lower extremities to the final outcome in sports competition and are critical for overall performance in swimming. It is generally accepted that in freestyle swimming, backstroke, and butterfly, over 85% of the thrust is produced by the arms [44]. Hawley and Williams [49] showed a positive correlation between upper-body anaerobic power, as measured by the upper-limb Wingate test, and performance in both sprint and middle-distance swimming events (400 m) [49] Based on these results, one can assume that HMWC supplementation in combination with a high carbohydrate diet may primarily improve results in swimming events between 50 and 400 m. Our study suggests that a shorter time to peak power for the upper limbs could improve speed in the first part of the sprint distance, where swimmers achieve the highest speed. It also suggests that these beneficial changes may influence the improvement in overall performance. This assumption is consistent with the improvement in the results obtained by the athletes in the Maglischo test. This test provides a protocol that allows the detection of changes in overall performance in swimmers and is commonly used to evaluate training effects in this group of athletes [30]. A higher rate of fatigue in the second lower-body Wingate test was a consequence of the greater peak power value compared to the first test, so the difference between peak power and minimum power was greater in the second test.

The oxidative stress that occurs in muscle cells under the influence of exercise stimulates transcription factors that are responsible for, among others, adapting cellular metabolism by making it more efficient. These include peroxisome proliferator-activated receptor-gamma coactivator (PGC-1 alpha), nuclear factor erythroid 2-related factor 2 (NRF2), nuclear factor kappa-light-chain-enhancer of activated B cells (NFkB), and mammalian target of rapamycin (mTOR) [57,58].

The improvement in the signaling pathways of reactive oxygen species (ROS)-influenced metabolic pathways resulting from regular physical activity leads to improved functioning of the cell’s energy metabolism, greater resistance to oxidative stress by strengthening antioxidant defense mechanisms, which translates into more efficient maintenance of the cell in a ROS-positive state. It also has a significant effect on reducing inflammation caused by physical activity. A small amount of ROS has a positive effect on the functioning of the cell, contributing to a more efficient metabolism, whereas an excess of ROS has a destructive effect on the whole cell. The best evidence for the health-promoting effect of a low pro- and antioxidant balance in favor of the former is the negative effect of too many antioxidants, e.g., in supplement form. Many scientific reports indicate that excessive antioxidant supplementation blocks the activity of ROS and even hinders the adaptation of the muscle cell to exercise. Most adaptive changes are specific to the type of muscle fiber [59,60].

Potential sources of ROS include the mitochondrial respiratory chain [61], but also the cytosolic pathway, which includes xanthine oxidase reactions as the final product of purine degradation [62] or the NADPH dehydrogenase reaction. Available results on oxidative modifications induced by anaerobic exercise [28] are limited and far fewer data are available on anaerobic exercise-induced oxidative modifications in elite swimmers. Recent evidence indicates that chronic anaerobic exercise training can induce adaptations that act to attenuate the exercise-induced oxidative stress and there is no specific exercise type effect in terms of redox balance [28,63,64,65,66]. Similar results, although under normobaric hypoxia conditions, were obtained by Poprzecki et al. [67]. The activities of blood antioxidant enzymes did not change in the hypoxic and normoxic group. The similarity in the obtained results is also reflected in the levels of MDA.

As the authors indicate, training under normobaric hypoxia is not an adequate stimulus for the excessive response of the antioxidant defense system, despite increased oxidative stress in these conditions. It is also worth mentioning that there are studies showing that the chlorine content in the air in the swimming pool may not be indifferent to the level of systemic oxidative stress [68].

A study investigating blood antioxidant status in road cyclists during progressive (VO_2_max) and constant cycling intensity tests (MLSS) demonstrated significant shifts in oxidative stress markers and antioxidant enzyme activities. These findings indicate that different exercise intensities can distinctly affect redox balance, reflecting the dynamic response of the antioxidant defense system during endurance exercise [69].

In this study, we focused, among others, on evaluating the efficiency of the defense mechanism against ROS by measuring the activity of antioxidant enzymes SOD, GR, CAT, and GPx, along with the low molecular weight antioxidant GSH. No changes in the activity of these physiological factors were observed in our study. These findings may be explained as a result of beneficial adaptive changes antioxidant enzymes induced by regular physical activity. Endogenous antioxidants cannot completely prevent oxidative damage. Increased ROS production caused by intense exercise may exceed the capacity of the antioxidant system, which in turn may lead to oxidative stress. That is why various types of supplements that strengthen the antioxidant response are so widely used in sports, which consist of an additional ergogenic aid to proper nutrition [70]. Professional athletes who train regularly for several years may reach a state of highest activity among antioxidant enzymes, the so-called “ceiling effect”. In line with this assumption, there is an insignificant difference between the baseline and achieved values after the Wingate test of these variables. Thus, one can suppose that the activities of this enzyme approached close to maximum values. On the contrary, upregulation in the activity of MDA, which is a marker of lipid peroxidation, was observed.

It is worth noting the increased concentration of uric acid (UA) in post-exercise samples after both T1 and T2 tests, and the FRAP values obtained. ROS are generated during anaerobic exercise mainly by the release of Fe ions into the plasma and the activation of xanthine oxidase, which produces UA as the final metabolite. Paradoxically, UA is considered to be the most important antioxidant due to its ability to chelate pro-oxidant iron ions. Souza-Junior et al. [71] investigated the relationship between UA and markers of oxidative stress in plasma after the Wingate test. Similar to our studies, he observed a 29% increase in UA during recovery with a concomitant lack of elevated MDA levels. He concluded that the increase in UA concentration was an auxiliary antioxidant response to oxidative stress after exercise (indirectly via iron), which is a well-known phenomenon, and the high correlation between total UA and FRAP in plasma only confirmed this hypothesis.

## 5. Conclusions

The present study demonstrates that the combination of a high carbohydrate diet and HMWC supplementation effectively enhances anaerobic performance in elite swimmers. Significant improvements were observed in peak power output and swimming velocity, indicating the potential of these dietary strategies to optimize short-distance performance. Notably, the benefits were more pronounced for lower limb performance in the Wingate test and for the latter stages of the swimming test, suggesting improved glycogen availability and utilization during high-intensity efforts.

However, the intervention did not induce measurable changes in antioxidant enzyme activities (SOD, CAT, GPx, GR) or low molecular weight antioxidants, indicating that these systems might already operate near maximal capacity in well-trained athletes. The observed increase in lipid peroxidation (MDA levels) post-supplementation underscores the challenge of managing oxidative stress during intense training. These findings suggest a potential trade-off between enhanced performance and increased oxidative stress, highlighting the importance of tailoring nutritional strategies to balance these effects.

However, overall conclusions are limited by the small sample size and lack of a control group. This research contributes to the growing body of evidence supporting the role of targeted carbohydrate supplementation in high-performance athletes. Future studies should explore the long-term implications of such interventions with a placebo and/or a control group, particularly the impact on redox homeostasis and recovery, to refine dietary recommendations for elite athletes preparing for international competition.

## 6. Practical Implications

Current research suggests that a one-size-fits-all approach may not be the most effective strategy due to individual differences in physiological responses to applied stimuli [72,73]. Based on these observations, it would be appropriate to consider revising dietary and supplementation strategies for each athlete individually—first during intensive training sessions and then during lower priority competitions. If successful, these tested strategies could then be implemented during the main competitions of the season.

A key advantage of carbohydrates is their safety. First, carbohydrate supplements are the products with the lowest risk of contamination with banned substances. Second, the only notable disadvantage in carbohydrate supplementation is a slight increase in body weight (2–3%), which does not seem to have a significant effect on swimming performance.

Based on our study, we can assume that athletes competing in swimming sprints and middle-distance events (50–400 m) may benefit from a high carbohydrate diet combined with high molecular weight carbohydrate (HMWC) supplementation. This strategy may increase peak power output, improve sprint speed, and facilitate glycogen availability for high intensity efforts. In addition, power- and sprint-focused mesocycles should include targeted carbohydrate loading to maximize glycogen storage and optimize anaerobic energy production. Coaches and sports nutritionists should tailor carbohydrate intake to training intensity and competition schedules to support energy requirements.

Although carbohydrate supplementation improves performance, it may also increase oxidative stress as indicated by elevated MDA levels. Athletes should focus on a balanced antioxidant intake from whole foods rather than excessive antioxidant supplementation, which may blunt adaptation.

The practical application of these findings extends beyond swimming, as the benefits of HMWC supplementation have been documented in several high-intensity sports. However, given the limitations of this study, including the small sample size and lack of a control group, further research is warranted to determine the long-term implications. Future research should include placebo-controlled designs to investigate the effects of carbohydrate supplementation on recovery, redox homeostasis, and sustained performance improvements in elite athletes preparing for major competitions.

## Figures and Tables

**Table 1:** Results of the Wingate test for upper and lower limbs before and after the dietary intervention.

		Test I (T1)			Test II (T2)			
		$\bar{x}$	SD	SE	$\bar{x}$	SD	SE	*d*
PP, W	UL	456.6	81.5	28.81	465.1	54.8	19.37	0.12
	LL	791.3	106.3	37.58	839.8 *	120.1	42.46	0.43
PP relative, W/UL	UL	5.73	1.07	0.38	5.78	0.62	0.22	0.06
	LL	9.9	1.29	0.46	10.44 *	1.38	0.49	0.40
Time to PP, s	UL	11.02	5.68	2.01	9.95 *	5.55	1.96	0.19
	LL	9.87	4.52	1.60	9.4	6.63	2.34	0.08
Rate of fatigue, %	UL	12.4	5.21	1.84	12	6.3	2.23	0.07
	LL	13.8	7.0	2.47	19.3 ^#^	8.1	2.86	0.73
TW, J	UL	3221.2	575.7	203.54	3601	1977.7	699.22	0.26
	LL	6025	2531.5	895.02	5849.3	3060	1081.87	0.06
Work to PP relative, J/UL	UL	40.4	7.8	2.76	44.8	24.3	8.59	0.24
	LL	75	29.8	10.54	72.5	37.1	13.12	0.07
Work to PP po Pmax, J	UL	8459.7	1559.7	551.44	8108.3	2265.7	801.05	0.18
	LL	13,714	3591.6	1269.82	13,974.3	4749.3	1679.13	0.06
W from PP relative, J/UL	UL	106.3	20.8	7.35	100.9	28.4	10.04	0.22
	LL	172	45.3	16.02	173.6	57.9	20.47	0.03
TW, J	UL	11,664.3	1607.1	568.20	11,709.9	934.7	330.47	0.04
	LL	19,739.1	1655	585.13	19,823.6	2311.3	817.17	0.04
TW relative, J/UL	UL	146.1	22.8	8.06	145.6	12.6	4.45	0.03
	LL	246.9	17.8	6.29	246	24.1	8.52	0.04
Mean Power, W	UL	388.8	53.7	18.99	390.4	31.1	11.00	0.04
	LL	658	55.1	19.48	660.6	77	27.22	0.04
Mean Power relative, W/UL	UL	4.88	0.75	0.27	4.86	0.42	0.15	0.03
	LL	8.24	0.59	0.21	8.21	0.82	0.29	0.04

*—rest vs. P max with *p* ≤ 0.001; ^#^—rest vs. P max with *p* ≤ 0.005; PP—peak power; UL—upper limbs; LL—lower limbs; $\bar{x}$—mean; SD—standard deviation; SE—standard error; *d*—Cohen’s d.

**Table 2:** Acid–base balance before and after the dietary intervention before and after the Wingate test.

			Men (M)							
			Test I (T1)			Test II (T2)			T1 vs. T2	
	Time		$\bar{x}$	SD	SE	$\bar{x}$	SD	SE		η^2^
LA, mmol/L	Rest	UL	1.53	0.30	0.11	1.59	0.32	0.11	NS	
		LL	1.53	0.30	0.11	1.59	0.32	0.11	NS	
	P max	UL	7.51 *	1.71	0.60	8.04 *	1.20	0.42	NS	
		LL	8.89 ^#^	1.38	0.49	9.38 ^#^	1.80	0.64	NS	
pH	Rest	UL	7.42	0.05	0.02	7.41	0.02	0.01	NS	
		LL	7.42	0.05	0.02	7.41	0.02	0.01	NS	
	P max	UL	7.30 *	0.05	0.02	7.30 *	0.05	0.02	NS	
		LL	7.28 ^#^	0.05	0.02	7.27 ^#^	0.05	0.02	NS	
SB, mmol/L	Rest	UL	25.10	0.70	0.25	23.90	1.20	0.42	*p* ≤ 0.05	0.05
		LL	25.10	0.70	0.25	23.90	1.20	0.42	*p* ≤ 0.05	0.05
	P max	UL	17.10 *	2.70	0.95	16.30 *	1.50	0.53	NS	
		LL	16.30 ^#^	1.80	0.64	15.60 ^#^	1.80	0.64	NS	

*—rest vs. P max UL with *p* ≤ 0.05; ^#^—rest vs. P max LL with *p* ≤ 0.05; NS—not statistically important; LA—lactate; SB—sodium bicarbonate; UL—upper limbs; LL—lower limbs; $\bar{x}$—mean; SD—standard deviation; SE—standard error; η^2^—eta-square.

**Table 3:** A comparative analysis of the mean swimming speeds of the athletes during the entire 8 × 100 m swimming test and on the final two sections of this test.

Speed [m/s]	T1			T2			
	$\bar{x}$	SD	SE	$\bar{x}$	SD	SE	*d*
V^mean^	1.483	0.08	0.03	1.481	0.07	0.02	0.027
V^7^	1.598	0.10	0.04	1.625 *	0.10	0.04	0.27
V^8^	1.670	0.11	0.04	1.709 *	0.13	0.05	0.32

*—T1 vs. T2 *p* ≤ 0.001; V—mean swimming speed; V^7^—mean swimming speed on the 7th section; V^8^—mean swimming speed on the 8th section; $\bar{x}$—mean; SD—standard deviation; SE—standard error; *d*—Cohen’s d.

**Table 4:** A comparative analysis of metabolic and biochemical variables in blood collected prior to and following the completion of the swimming test before (T1) and after (T2) the supplementation intervention.

Time		Men (M)							
		Test I (T1)			Test II (T2)			T1 vs. T2	
		$\bar{x}$	SD	SE	$\bar{x}$	SD	SE		η^2^
Glucose, mg/dL	Rest	80.00	10.30	3.64	80.00	11.50	4.07	NS	
	After	97.80 *	10.10	3.57	102.00 *	9.10	3.22	NS	
LA mmol/L	Rest	1.53	0.27	0.10	1.28	0.20	0.07	NS	
	After	10.46 *	2.06	0.73	10.38 *	1.63	0.58	NS	
UA, mg/dL	Rest	4.99	0.68	0.24	5.34	0.39	0.14	NS	
	After	6.69 *	0.97	0.34	5.01	1.03	0.36	*p* ≤ 0.05	0.10
pH	Rest	7.39	0.03	0.01	7.40	0.02	0.01	NS	
	After	7.17 *	0.07	0.02	7.18 *	0.06	0.02	NS	
SB, mmol/L	Rest	23.90	1.70	0.60	24.50	0.80	0.28	NS	
	After	12.00 *	2.50	0.88	11.90 *	2.00	0.71	NS	
BE, mmol/L	Rest	−0.60	1.90	0.67	0.10	1.00	0.35	NS	
	After	−17.20 *	4.00	1.41	−17.20 *	3.20	1.13	NS	
GPx, U/g Hb	Rest	46.20	9.60	3.39	34.80	5.60	1.98	NS	
	After	44.70	7.10	2.51	36.60	7.40	2.62	NS	
SOD, U/g Hb	Rest	1500.20	243.50	86.09	1112.90	481.70	170.31	NS	
	After	1560.90	239.50	84.68	1113.60	492.30	174.05	NS	
GR, U/g Hb	Rest	22.30	6.10	2.16	20.90	4.90	1.73	NS	
	After	22.70	7.70	2.72	20.70	8.80	3.11	NS	
CAT, k/g Hb	Rest	167.90	46.20	16.33	185.40	45.60	16.12	NS	
	After	205.30	52.60	18.60	187.50	53.90	19.06	NS	
GSH, μg/mg Hb	Rest	2.4	0.8	0.28	2.5	0.5	0.18	NS	
	After	2	0.6	0.21	2.7	0.4	0.14	NS	
CK, U/L	Rest	96.8	39.1	13.82	211.7	93.1	32.92	*p* ≤ 0.01	0.14
	After	164.2 *	101.7	35.96	236.4 *	100.7	35.60	NS	
LDH total, U/L	Rest	240.5	54.1	19.13	247.2	79.6	28.14	NS	
	After	299.5	47.6	16.83	272.9	47.1	16.65	NS	
FRAP, μM Troloxu	Rest	867.3	118.2	41.79	814	51.7	18.28	NS	
	After	988.5	139.2	49.21	816.5	69.5	24.57	*p* ≤ 0.05	0.13
MDA, nmol/mL	Rest	2.41	0.9	0.32	4.97	2.1	0.74	*p* ≤ 0.05	0.12
	After	2.72	0.91	0.32	5.3	2.37	0.84	*p* ≤ 0.05	0.06

*—rest vs. after *p* ≤ 0.05; NS—not statistically important; LA—lactate; UA—uric acid; SB—sodium bicarbonate; BE—base excess; GPx—glutathione peroxidase; SOD—superoxide dismutase; GR—glutathione reductase; CAT—catalase; GSH—reduced glutathione; CK—creatine kinase; LDH—lactate dehydrogenase; FRAP—ferric-ion-reducing antioxidant power; MDA—malondialdehyde; rest—before the test; after—after the test; $\bar{x}$—mean; SD—standard deviation; SE—standard error; η^2^—eta-square.

## Data Availability

The original contributions presented in this study are included in the article. Further inquiries can be directed to the corresponding author.
